# Health and equity impacts of global consultancy firms

**DOI:** 10.1186/s12992-024-01061-9

**Published:** 2024-07-25

**Authors:** Julia Anaf, Fran Baum

**Affiliations:** 1 North Tce Campus, Stretton Health Equity, Stretton Institute, University of Adelaide, Adelaide, 5005 Australia; 2 Health Equity, North Tce Campus, Stretton Institute, University of Adelaide, Adelaide, 5005 Australia

**Keywords:** Health, Equity, Global consultants, Neoliberalism, Taxation

## Abstract

**Background:**

Concern is growing over the power, influence, and threats to health and equity from the operations of large global consultancy firms. Collectively, these firms support a neoliberal policy environment promoting business interests ahead of public health. Global consultancy firms act as commercial determinants of health, an evolving area of research over recent years. However, this research mainly focuses on specific corporations or industry sectors, especially those which produce harmful products, including ultra-processed food, alcohol, and fossil fuels. It is therefore important to expand the focus to include large global consultancy firms and place a public health and equity lens over their operations.

**Main body:**

Global consultancy firms have wide-ranging conflicts of interest. These arise from the ‘revolving door’ employment strategies between their own staff and those from government and regulatory bodies. These firms also advise governments on taxation and other matters while concurrently advising corporate clients on ways to minimise taxation. They advise fossil fuel corporations while also advising governments on climate and health policies. These firms undermine the capabilities of the public sector through the outsourcing of traditional public sector roles to these private interests. Consultancy firms foster private interests through their engagement with the higher education sector, and thereby weaken the tradition of transparent management of university affairs by accountable university councils. While private consultancies cannot be blamed for all the negative consequences for health and equity caused by the problems associated with globalisation and advanced capitalism, they have played a role in amplifying them.

**Conclusion:**

Addressing the negative impacts of global consultancy firms will require strengthening the public sector, enforcing greater transparency, accountability, and minimising conflicts of interest. It will also demand critical thought, counter discourses, and activism to reframe the narratives supporting neo-liberal ideas of governance that are promoted in both government and business arenas.

## Background

Concern is growing about the power, influence, and threats to health and equity from the operations of large global consultancy firms [1–4]. These firms promote neoliberal ideology and practices that advocate for small government and free market capitalism and so are enablers of the operation of trans-national capital particularly Trans-national Corporations (TNCs). They implement the tenets of neo-liberalism through their wide-ranging global operations. One major tenet is that the private sector is inherently more cost-effective and efficient than the public sector, with large consultancy firms capitalising on this view and supporting New Public Management practices over recent decades [5, 6].

Although numerous global companies offer similar forms of advice worldwide [7], the four largest professional services firms (the ‘Big Four’) are Deloitte, Ernst & Young (EY), Klynveld Peat Marwick Goerdeler (KPMG), and PricewaterhouseCoopers (PwC). They offer a wide range of services including auditing and taxation advice to private entities and governments [8]. They camouflage their broad financial interests through discursive appeals to the common good, public interest, national economic performance, and their technical expertise [8]. The ‘Big Four’ hold the dominant share of the global accounting and auditing industry with a combined revenue of US $200Billion [9] and more than 1,000,000 staff globally [10].

The three largest global consultancy and strategy firms (the ‘Big Three’), measured by revenue, are McKinsey and Co, Boston Consulting Group, and Bain and Co. Their work focuses on general strategy, organization, marketing, and operations. Together they hold revenue of approximately $US 25.5 Billion and employ 65,500 staff [11].

Collectively, these firms support the *status quo* on global and national regulations which reflect a neoliberal policy environment promoting business interests ahead of public health and wellbeing [12]. They have been prominent actors in establishing the post-1970s neoliberal hegemony, and have benefitted from the financialization of the economy [13]. These firms have also become skilled at manipulating rules to promote their own financial interests [4, 14, 15]. Being deeply entwined within private financial sector networks, they maintain close ties with financial lobbying groups [5, 16]. They are part of capitalism as an overarching global and historical structure that produces and co-produces transmission of ill health and disease. These transmitters include transnational capital, poverty and inequality [17]. Material interests are fostered by an institutional and ideological complex which intensifies and maintains the externalities of capitalism on human health [17].

Together, consultancy firms act as commercial determinants of health (CDoH); being part of the ‘systems, practices, and pathways through which commercial actors drive health and equity’ [18, 19]. The conceptualisation of CDoH as detrimental to health [19, 20] differs from more neutral framing of social determinants of health (SDoH) in which conditions are recognised for their potential as either protective or risk factors [20]. Definitions of CDoH reflect different interpretations of ‘health’ in which a biomedical focus can prioritise non-communicable diseases above other social, environmental, legal, economic, and ethical drivers that affect health. A focus on the damaging products produced by commercial actors and their associated activities can limit the definition of CDoH to unhealthy commodities and transnational corporations (TNCs) without considering the broader array of commercial actors.

More nuanced approaches recognise the complexity of CDoH as a composite of risk factors which allows clearer identification of relative vulnerabilities by specific populations over time and place, and across other variables including age, gender, and socioeconomic status [21]. Thus, CDoH should be conceptualised more broadly to emphasise the many ways by which inequality and human health are influenced by commercial factors.

The health and equity impacts of CDoH have come into stark relief in recent years, but to advance scholarship, and a greater understanding of the health and equity impacts of global consultancy firms, it is critical to apply a public health and equity lens to their operations. A health and equity lens, grounded in human rights, considers systemic social and health inequities and the SDoH. Health equity is a process of removing structural patterns or social and economic barriers to health that drive health disparities [22]. Applying this lens involves understanding perceived and actual conflicts of interest within these firms’ global operations that impact negatively on health and equity. In compiling the material and reviewing the literature on the health and equity impacts of these firms, the authors used keyword search terms including health and equity strategically to help identify appropriate resources.

## Conflicts of interests within global operations

Conflicts of interest (COIs) are the set of circumstances which create risks that professional judgement or actions concerning a primary interest will be unduly influenced by a secondary interest [23]. This is particularly important for public health and health promotion, as firms with COIs impinge on the key mandates of the state institutions which are charged with maintaining and protecting public health [24, 25]. These COIs arise within the context of capitalism as an overarching global phenomenon. Super-structural factors and forces including economic and market ideologies, regimes, and rules also determine health outcomes and life chances [17]. This system has fostered the global growth and spread of TNCs which are key clients of global consultancy firms [17], often creating a symbiotic relationship between them.

Consultancy firms may have COIs which arise from the ‘revolving door’ between their staff, and those of revenue authorities, government departments, and corporate regulators. This potentially facilitates inappropriate or unethical exchange of information [26, 27]. Government regulators may also face COIs between a perceived need to litigate against these firms due to legal or ethical issues, and concerns about causing an even greater concentration of power in the hands of fewer firms. These entities can be understood to be ‘too big to fail’ and ‘too concentrated to indict’, which potentially affords them a ‘strange form of legal immunity’ [28]. In most professional settings, personnel including Boards of Directors, university staff applying for research ethics approval, and public servants, must declare COIs, but these firms hold lower levels of accountability. The increased use of private firms in the public sector is seen to represent ‘a solvent dissolving the boundary between public and private interests’ [29], or even a ‘shadow public service’ [30, 31].

### Conflicts of interest and consultancy firms

Wide-ranging COIs arise from these firms’ roles as professional auditors, while also consultants and advisers on taxation matters, government contracts, and the outsourcing of government functions [1]. One such example arises when ‘Big Four’ firms advise governments on taxation matters, while concurrently offering advice to their corporate clients on ways to minimise their taxation liabilities [32–34]. This includes advice on using variable tax rates across different jurisdictions through transfer pricing [1]. As confirmed by exposés from ‘whistleblowers’ who informed the 2017 ‘Paradise Papers’ [35], the 2015 ‘Panama Papers’, and the 2014 ‘Luxembourg Leaks’ on global tax evasion [36], income is moved to low-tax locations or ‘tax havens’ [37]. Paper losses are generated to allow clients to benefit from favourable tax assessment of depreciation or debt [38].

These actions are facilitated by a lack of global taxation regulations. Together with exploiting existing laws, the ‘Big Four’ firms have become important actors in law-making. They are a key source of high-level expertise and are extensively represented in national policymaking [39]. These firms are both a symptom of poor regulation and enforcement, and a mechanism by which poor behaviour is condoned. They therefore act as CDoH, broadly defined.

COIs also exist between these firms’ legally independent or investigative roles such as auditing, and broader supportive or advisory roles. This conflict is sometimes termed ‘walking both sides of the street’ [1]. The clear COIs inherent in these roles had led to calls for these functions to be legally separated operationally [40, 41]. Such COIs may also cause firms’ hesitancy to ask ‘difficult’ questions, or rigorously investigate client records that may uncover fraud, as this could undermine lucrative relationships. They can also lead to undermining ‘frank and fearless’ advice from public servants [42].

Despite the potential for COIs to threaten fair public sector governance, consultancy firms’ advice to governments also includes the design of tax policy; sometimes involving secondment of staff to help draft legislation. They may thereby potentially develop strategies to assist their clients to minimise taxation liabilities [43]. These firms advocate privately and publicly, both nationally and internationally, for specific policy changes in fora including the OECD [39].

Taxation authority staff maintain that ‘Big Four’ firms are more likely than local accounting firms to advise clients to use aggressive taxation strategies. An Organization for Economic Co-operation and Development (OECD) report [44] on government officials’ perceptions of the behaviour of these firms deemed that although formally cooperative, they fail to follow the ‘spirit’ or intention of the law [45]. The firms are largely unaccountable and unregulated; penetrating governments at all levels [32]. The partnership model on which these firms are based means they are not required to audit their accounts or release them publicly. They are not subject to the Corporations Act and corporate taxation [46].

Lack of compliance and dishonest conduct result from these firms’ range of COIs [33], and cost governments and taxpayers an estimated $US I trillion per annum globally by 2016 [32]. This foregone revenue may otherwise allow for much greater health and social investment by nation states. This would enable them to support better health, education, and welfare services, and to undertake measures aimed at reducing health inequities.

### Conflicts of interest: consultancy and strategy firms

Conflicts of interest also arise for global consultancy and strategy firms such as the ‘Big Three’ [47, 48]. One indicative example is a ‘Big Three’ firm, McKinsey, advising multiple companies within a sector, thus having capacity to exchange their confidential information. This was while concurrently advising the regulators overseeing these same companies [4]. The same firm also advised health-care providers on ways to address the negative health impacts from smoking tobacco, while also advising the responsible body for regulating tobacco corporations. A further example of COIs is that the firm has advised nearly every major pharmaceutical company, as well as the government regulators who monitored them [4].

A further indicative example of consultancy and strategy firms’ COIs is another ‘Big Three’ firm, BCG, being implicated in the failure of the United Nations Framework Convention on Climate Change (UNFCCC) to properly address undue influence, and to manage COIs within the negotiation process. While providing advice to at least 19 of the world’s 25 largest oil companies, this firm also had a lucrative contract to provide strategy implementation advice for the UK Government’s 26th UN Climate Change Conference of Parties (COP 26) Unit [49].

Eckl & Hanrieder [50] present a case study of a consultant involved in a WHO reform and note three practices in which these consultants engage. These are: curating voices and input (including their own) into reform packages; promoting content compatible with their values; and engaging in self-effacement practices that undermine accountability to stakeholders. This results in certain actors being excluded from the reform process and so being disempowered.

More generally, consultancy firms may make self-interested recommendations to governments, their agencies, and even the university sector on the unstated basis that it may thereby generate further work for the firm; sometimes referred to as ‘land and expand’ strategies [51].

### Conflicts of interest and the university sector

Consultancy and strategy firms also engage with universities which, over recent decades, have been transformed from public-good institutions to ones that largely mimic the hierarchical corporate operations of the commercial sector [52]. This is despite universities also being largely public institutions with public functions, funded by taxpayers, and transparently managed by a council which is accountable to both the university and the broader electorate [52]. Consultancy firms’ roles span student counselling, IT securitisation, and in-service training, whereby expert in-house professional staff are replaced with companies which are answerable to their shareholders, not to traditional university stakeholders [53]. Their roles also include ‘helping’ universities with board governance, mergers, acquisitions and alliances, strategies and organisation [54]. They co-design curricula and oversee restructuring. The Deloitte University for Europe, the Middle East, and Africa opened in 2013: one of seven Deloitte universities worldwide [55].

While links between business and universities have some benefits, they also pose health threats to university staff and the population at large [56]. The corporatisation of universities, including constant change and restructuring facilitated through external advice (eg [57]), can result in undermining the protection of tenure which allows academics to speak out ‘without fear or favour’ in support of the public good, which is part of their role [56]. The scope of these firms’ operations highlights the COIs between public and private interests in higher education, and weakening of the tradition of transparent management of university affairs by accountable university councils [52]. The ‘intrusion of a market ideology into the heart of academic life’ is seen to be the single greatest threat to the future of universities ([58], p.x).

There is now great interest from university graduates to gain employment with the ‘Big Four’ firms, rather than using their skills to promote the public interest in the public sector. An analysis of the top 60 UK universities, calculated as a proportion of each university’s enrolment size, based on student enrolment for the 2020/21 academic year, revealed the total number of graduates currently employed in these firms. The highest number were graduates from the London School of Economics (5776), and Cambridge (3401) [59, 60]. The growing marketisation of public services has also led to these firms ‘poaching’ the best and most experienced public servants, thereby reducing state capacity [61]. The ‘Big Three’ firms with a vested interest in the private sector also attract great employment interest from graduates [62].

## Global health and equity impacts from firms’ operations

The values and operations of large consulting firms have consequences for public services across the world, and do not support a focus on reducing inequity through public policy. A vast literature documents improper practices by global professional services and management firms [1, 4, 36]. The health and equity impacts from the indicative examples of COIs exacerbate the loss of public trust in governments. Trust is imperative to ensure greater compliance with policies including public health responses, taxation systems, and regulations that promote public health, equity, and the broader public interest. Trust strengthens social cohesion, political participation, and builds institutional legitimacy [63].Thus, any threat to government and to other key institutions from loss of trust and legitimacy is a growing concern [64, 65].

### Corruption and deception

Corruption is a serious threat to global health, leading to financial waste and adverse health consequences. An estimated $455billion of the $7.35 trillion spent on health care globally each year is lost to fraud and corruption [66]. Corruption is a complex and multifaceted challenge. Offenses range from smaller-scale acts including absenteeism and informal payments, to much larger scale corruption at administrative levels. It reflects the interplay between inadequate public funding, a burgeoning unregulated role of the private sector, and lack of overarching transparency in governance [67].

One example was highlighted by an inquiry into South Africa’s largest post-apartheid corruption scandal as a clear example of how the private sector colluded with the breakdown of public institutions. It was revealed that one ‘Big Three’ firm had assisted in undermining the country’s revenue service in ways that would facilitate corruption [68, 69]. Corruption especially damages poor and disadvantaged populations by reducing state resources for social spending. It may distract the allocation of public resources to the public good by distorting decision-makers’ incentives, and by diverting public spending towards more lucrative projects [70].

The relationship between consultancy firms and governments may also be used strategically, rather than as a basis for adopting sound public policies that promote public trust and equitable population health and wellbeing [71]. This issue came to light in the recent Royal Commission into the Australian ‘Robodebt’ scheme involving an unlawful method of automated debt assessment and recovery. This was employed by the Australian Government against welfare recipients who were already in vulnerable circumstances [72]. The scheme led to documented cases of suicide, financial hardship, and feelings of hopelessness and other mental health impacts [72]. It has been described as ‘scripting lives of disempowered suffering instead of supporting safety, work, families, and care in our country’ [73]. The consultancy firm PwC was a party to the scandal. The firm was cited in the Royal Commission for their strategic and critical lack of detail, albeit condoned by government, when reporting important material issues, as part of their contract to evaluate the program. This contributed to the adverse impacts including health for welfare recipients [74].

There is a vast literature on corporate social responsibility (CSR) including its potential for deception [75–77]. At its most basic and earliest understanding, CSR is employed by companies to ‘give something back’ to the community to benefit the public good [78]. However, negative impacts may arise from consulting firms offering advice to multinational corporations on how to apply CSR practices strategically. This can be used to promote their social licence to operate, in ways that are likely to shift negative, albeit potentially warranted, public perceptions of a corporation or industry that has harmful impacts from their products or operations. This corporate ‘washing’ or deception also applies to firms ‘providing independent evidence to win public policy debates’ for vested interests, rather than for promoting public health and equity [79].

The ‘Big Four’ firms state the importance of good corporate citizenship and highlight their own CSR initiatives on their websites. These focus on ‘building trust’ [80], ‘acting responsibly’ [81], ‘promoting highest levels of ethical behaviour’ [82] and ‘commitment to our communities’ [83]. However, many examples of breaches of such commitments are found in the literature. These include EY in the US facing the largest historic fine for withholding evidence about the discreditable conduct of staff cheating in examinations [84]. This is despite the prime role of auditors being complete allegiance to the public good. Such failures question professional integrity and the very culture that should promote ethical behaviour in the public interest [85].

Deloitte has been fined for failures in their auditing practices [86] and, in what the Australian treasurer termed a ‘grave breach of trust’, a former PwC tax partner shared confidential treasury information on multinational companies’ taxation reform with other staff and partners [87]. This had the potential for using privileged information to prioritise private financial interests over public interests. KPMG is known to have forged documents and misled the UK Financial Reporting Council over its auditing of a range of companies [88] and was implicated in a corruption scandals in South Africa [89], as was McKinsey and Co [90]. These indicative breaches support the claim that CSR may be used as a tool to cynically ‘deflect attention and whitewash tarnished reputations’ [19]. Although some CSR efforts have real and meaningful effects, and can be used in good faith, often they contribute more to reputation building than to providing real benefits for society, especially due to these incentives being tax deductible ways to shape policy outcomes that can work against the public good [18].

### Impacts on employment and human rights

Employment is a critical determinant of health and equity [91]. Past scandals involving global firms at times of financial and other crises have revealed COIs and accounting failures that led to thousands of job losses, and company ‘bail outs’ by taxpayers worth billions of dollars [92]. Prior to the global financial crisis (2007–2008), failing banks were provided with unjustified positive financial assessments by their auditors [43]. Many low-income and marginalised people faced the greatest burdens from the subsequent financial and job losses based on this incorrect information [93].

Operations of global firms also have implications for human rights and democracy. At the time of the 2014 unofficial People’s Referendum on universal suffrage in Hong Kong, the ‘Big Four’ companies jointly devised advertisements in Hong Kong newspapers to oppose the referendum, claiming that protests would lead to the flight of multinational companies from Hong Kong, (presumably affecting their income) [94]. Although it is argued that in many other organisations subversion of human rights would be considered a badge of shame, one claim is that ‘at major accountancy firms it is increasingly considered to be a sign of business acumen’ [94].

In low and middle income countries, human rights, health, and equity may be particularly undermined as governments often face more severe governance challenges, limited finances, and have weak public sectors [95]. Therefore, issues of malfeasance by large global firms can be devastating for the people who face the harms associated with firms strategically but incorrectly ‘signing off’ on audits [95]. While the global population suffered negative outcomes during the global financial crisis, the ‘Big Four’ received massively increased levels of income from providing financial services. However, they also helped to design the range of financial products that had negatively affected individuals’ and companies’ balance sheets [92]. Neoliberal reforms within the healthcare sector using the services of global consultancy firms have been criticised in respect of PPPs for hospitals as being more expensive than if procured through traditional avenues [96, 97].

### Fostering wage inequities

There is evidence of the global impacts on health and equity from consulting firms fostering increased wage disparity. From as early as the 1950s, staff at McKinsey proposed ways in which CEOs could be better rewarded financially through incentive schemes linked to profits. This resulted in directors of Fortune 500 companies receiving an increased pay differential between their roles and those of production workers. In 1950, the chief executive of a traditional large company earned 20 times that of a production worker, By 2020, CEOs earned at least 351 times as much [4].

A large literature examines income inequality in relation to health, finding that health tends to be worse in more unequal societies [98]. Since 1990, income inequality has increased in most low-income countries and in some middle-income countries, with 71 per cent of the world’s population living in countries where inequality has increased [99]. Economic inequality has both physical and psychological consequences. More unequal societies have poorer interpersonal relationships, with close correlations between social inequality and mortality, lower life expectancy, drug use, increased incarceration increased, mental illness, and lack of trust [100].

### Consultancies and global health institutions

Management consulting firms have become ubiquitous in global health institutions and in countries in crises, with Boston Consulting Group and McKinsey the most well-known [101]. Conflicts of interest occur when these firms operate using ‘land and expand’ strategies. These include offering pro-bono work to health institutions in anticipation of further lucrative engagements with the original institution or in the wider health-related field. Consulting firm revenues have both public and private sources, including public–private partnerships and philanthropic institutions such as the Bill and Melinda Gates Foundation (BMGF). Both McKinsey and Boston Consulting Group have received lucrative contracts from BMGF, underscoring ‘the alignment in vision and approaches between management consulting firms and philanthro-capitalist ventures [101]. These empowered private actors, which do not face democratic oversight, wield great influence over global health policy, often promoting privatised solutions [102] and the use of global consultancy firms.

McKinsey and Co, the world’s largest management firm, has been involved in responses to major international disease outbreaks in recent decades including the Mers, Zika, and Ebola epidemics across different jurisdictions. Both McKinsey and BCG worked at Gavi, the Vaccine Alliance, and the Global Fund (both PPPs), UNITAID, the WHO, the Gates Foundation, and the global health nonprofit Partners in Health [103]. Vox (2019) reported that Gates has “spent more than $300 million on McKinsey and BCG between 2006 and 2017, according to the foundation’s tax returns. That’s more than the domestic health budget for an entire low-income country, like Haiti” [104]. The growing reliance on mainly US consulting firms is a symptom of a larger problem at global health organisations. Work is still undertaken by people in high- income countries for people in low and middle- income countries; often without their participation, imposing neoliberal public policy ideas within these jurisdictions, without a clear focus on the value of Comprehensive Primary Health Care (CPHC) [103, 105].

### Direct involvement in the health sector

McKinsey and Co’s advice has been linked to some of the most critical health crises over the last 50 years, including their role in the opioid epidemic which, since 1999, has taken the lives of almost one million people in the USA [103]. (See case study Table 1). In Australia, as in other global jurisdictions, McKinsey and other large global consultancy firms including EY, PwC, received multi-million-dollar contracts linked to the COVID-19 pandemic response without clear transparency and accountability [106–109].

**Table 1:** McKinsey’s role in the opioid crisis

Findings from the release of 114,000 documents from The https://www.industrydocuments.ucsf.edu/opioids/Opioid Industry Documents Archive (OIDA) revealed how McKinsey advised Purdue Pharma, Endo Pharmaceuticals, Johnson & Johnson and Mallinckrodt, all opioid makers, to help boost sales, despite increasing public concern over a growing opioid epidemic. McKinsey received US 83.7m dollars in fees from the pharmaceutical company Purdue for marketing advice to bolster the sale of the pain-relieving drug OxyContin which fuelled the opioid crisis [110]. The consultancy had been described as a ‘machine that… destroyed lives’ by targeting doctors whom they knew would over-prescribe. McKinsey also earned millions of dollars by assisting other firms to develop similar sales and marketing plans [111]. Its client Purdue also admitted to facilitating the supply of drugs ‘without legitimate medical purpose’, paying doctors and others illegal ‘kickbacks’ to prescribe the drugs [111]. McKinsey not only advised Purdue on strategies to boost the drug’s sales but also how to ‘burnish its image’, including countering ‘the emotional messages’ of mothers whose children overdosed [112]. McKinsey eventually paid a US $573 million legal settlement over its role in the crisis, which helped to fund drug treatment and other efforts to address the negative health impacts [113]. A statement on the consultancy’s website maintains ‘Our past work for opioid manufacturers, while lawful, fell short of the high standards we set for ourselves… We have also reached agreements with political subdivisions, school districts, Native American tribes, and third-party payors. The firm entered into these agreements, which contain no admissions of liability or wrongdoing, to avoid the time and expense of protracted litigation’ [114].	

The role of McKinsey and Co is also documented in a case study of the negative impacts that can occur from a consulting firm’s engagement in global health institutions (See Table 2). It is a reminder of the need to monitor these partnerships across a range of large global consultancy firms and gather data on more recent experiences.

**Table 2:** Failure of UNITAID funding scheme

UNITAID, created in 2006, makes new health products available and affordable for people in low and middle-income countries. It was universally acclaimed for establishing a new sustainable funding stream through a small tax on airline tickets in certain countries, generating close to US$ 300 million in revenues in its first year of operations. However, by 2007, finding new countries with significant airline markets was proving much harder than anticipated. Chairman, Philippe Douste-Blazy and allies in the tourism business suggested the alternative of working directly with the industry, offering travellers the option of voluntary micro-contributions. Interested board members suggested that a feasibility study be conducted to validate the approach and add credibility. McKinsey & Company estimated that it could undertake the three-month study for US$ 1 million. Despite the cost, the Chairman was convinced that “it would be easier to get the UK, the Bill and Melinda Gates Foundation (BMGF) and the WHO to sign off if such a document existed.” The BMGF had been contributing to UNITAID from its inception through funding McKinsey’s involvement and agreed to financially support the initiative’s modelling and development of its business plan. The McKinsey study suggested that the new mechanism could raise between US$ 500 million and US$ 1 billion annually from private sources within five years, almost doubling UNITAID’s budget from the airline-ticket tax and other contributions. The Board endorsed the plan and allocated a provisional budget of US$ 9 million for the first year, and US$ 12 million for the second year. A director was appointed to set up the Millennium Foundation to host the project, and the team rapidly grew under the name Massive Good. However, by July 2010, the foundation had fallen drastically behind schedule, raising less revenue than what had been granted by governments for salaries, advertising, and legal expenses. The Board decided that the initiative should be independently assessed by another firm Dalberg
The impact of the recommendations made by management consultants is rarely, if ever, evaluated which is particularly ironic given how often consulting firms are invited into the health sphere based on claims of increased impact or value for money. And paradoxically, governments and bilateral donors, otherwise committed to transparency and accountability, often overlook these considerations when they employ consultants. At the country level, the situation is rarely much better as large consulting firms have no formal commitments to the countries in which they work, connection with communities, or political accountability. The story of Massive Good had faded from the public eye by 2012, offering a telling example of the gap between the rhetoric of efficiency gained through management consulting advice and the reality, and the lack of accountability surrounding consulting firms’ engagement
In December 2012, UNITAID’s chair reported that the Millenium Foundation had failed to reach its objectives and it was dissolved with the loss of millions of US dollars. However, McKinsey and its consultants were never held accountable for the loss of public resources, in which their work played a large role. The UNITAID Board itself remained mostly silent about the incident, mindful of its own failure to appropriately scrutinize the proposal and oversee the efforts. While it might have had an opportunity to demand accounts, it chose not to, unwilling to create further backlash against funding for health, and expose UNITAID to the types of criticism for wastefulness once levelled against traditional health institutions from which they were supposed to differ

Adapted from People’s Health Movement Global Health Watch 5 (2017): Box D3.2 “UNITAIDS funding scheme: ‘massive’ failure”

## Discussion

*A weakened state structure is like a flagging army; the commandos—i.e. the private armed organizations enter the field and they have two tasks: to make use of illegal means, while the State appears to remain within legality, and thus to reorganize the State itself [* 115 *].*

While global consultancy firms cannot be blamed for all negative consequences for health and equity that are caused by the problems of globalisation and advanced capitalism, they have played a role in amplifying them. As Stein contends, consultants work on social relationships as they are often engaged to speed up corporate activities and thus act as one of the drivers of capitalist acceleration [116]. Consequences include the ‘hollowing out’ of the capacity of public institutions, the climate crisis, growing inequality, and the financialization of economies. These powerful and influential firms have both benefitted from, and have helped to shape this milieu [1]. For example, the European Commission’s advisory group members investigating transfer pricing as part of taxation strategies were all selected from multinational corporations and the ‘Big Four’ firms. No NGO or academic representatives, who could promote the views and interests of public health, and other professionals working for social justice and human rights were nominated to this body [117].

The oligopolistic form of large auditing services leads to government regulators being hesitant to litigate against any of these firms, with concerns that this would result in an even greater concentration of power. Governments and the public services have become excessively dependent on consultants, at the expense of frank and fearless advice and state capacity [1, 118]. The often symbiotic relationship between global consultancy firms and governments means that unwelcome expert knowledge and advice can be ignored, used selectively, or be commissioned to either reflect or reinforce entrenched positions. Public servants can be bypassed to instead implement the choices of corporate lobbyists. This can lead to policy paralysis and entrenching narrow business interests [119].

### Taking action for health and equity

Global professional services and consultancy firms are CDoH [120]. While no single intervention can successfully address the negative aspects of CDoH [121], in respect of global professional services and management firms, there is a strong argument for the following set of regulatory and taxation principles suggested by Rozvany (Table 3) (cited in [122]) being applied.

**Table 3:** Suggested regulatory and taxation principles for global consultancy firms

**Regulatory Principle 1:** All jurisdictions should ensure that an accounting or professional services firm or organization does not provide both taxation and audit services to the public
**Regulatory Principle 2:** All jurisdictions must ensure that there is sufficient competition in both taxation and audit services to provide an orderly market
**Taxation Principle 1:** All jurisdictions should encourage ethical tax behaviors by way of economic incentives through discounts in the corporate tax rate or other real incentive measures
**Taxation Principle 2:** All jurisdictions should ensure that appropriate punitive measures reflecting the fraudulent nature of aggressive taxation behaviors be implemented
**Taxation Principle 3:** All expenses originating from a jurisdiction internationally characterized as a tax haven will be denied a tax deduction in the home jurisdiction Implementation of these principles will increase the propensity of governments to use the increased taxation for purposes which promote population health and reduce health inequities

Other active responses have been proposed. For example, rebuilding the capability of public sector organisations that have been ‘hollowed out’ through contracting services to private firms, to function as economic value creators rather than as wasteful and inefficient value extractors. The resilience of ‘global’ health governance largely depends on what happens at the national and sub-national levels [102]. To support innovative policy advice, governments can engage with research institutions in genuine partnerships from which the public sector can receive advice that is independent of commercial interests. Public servants must once again be able to provide ‘frank and fearless’ advice to government [123] including in relation to how policy can be used to reduce inequities and ensure it is furthering the public good. Using policy in this way requires consistent and focussed strategic thinking which is much less likely to happen when policy work happens in silos through contracted out disjointed pieces of work.

Eckl and Hanrieder [50] recommend “new standards for engagement and transparency, including proper documentation and public accountability of WHO reform consultants”, and Corruption Watch has called for regulation of large private firms doing business with the state [124]. Addressing firms’ COIs will require enforcing greater transparency and accountability [125], including for university executives and senior managers [119].

There are no rules mandating consulting companies to disclose information about for whom they work, and Mazzucato and Collington [126] argue that this must be rectified. Strong action must be taken against breaches of COI management, as well as improved ‘whistleblower’ protection [125] due to the power disparity between corporate entities and ‘whistleblowers’ acting in the public interest. To mitigate the risks related to consultancies providing services *pro bono* or below market rates in ‘land and expand’ tactics, public servants should calculate in advance the appropriate economic value for the service while still seeking a competitive price [126]. Adopting these measures will begin to address these firms’ negative health and equity impacts.

## Conclusion

The growth of private consultancies has corroded democracy by ‘hollowing out’ public sector capacity. This contributes to government decision-making that is less likely to be in the public interest, given that consulting firms have evident COIs which are frequently incompatible with those of public health and equity. Consultants are poor substitutes for ‘frank and fearless’ public servants. The use of global consulting and professional services firms should be strongly circumscribed, with enforced rigorous accountability. Dealing with the negative health and equity impacts from the operations of these firms will require critical thought, counter discourses, and activism. This is to reframe the narratives supporting neo-liberal ideas of governance that are promoted in both government and business arenas.

## Data Availability

Not applicable.
